# New Appliances and Things Medical

**Published:** 1899-03-04

**Authors:** 


### NEW APPLIANCES AND THINGS MEDICAL.

**[We shall be glad to receive, at our Office, 28 & 29, Southampton Street, Strand, London, W.C., from the manufacturers, specimens of all new preparations and appliances which may be brought out from time to time.]**

### CARTER'S BREAKFAST OATS, "GOLDEN WING," BRAND.

(James Carter and Co., 237 and 238, High Holborn, London.)

The "Golden Wing" oats are particularly well adapted for conversion into a readily made porridge; further than this, excellent scones, puddings, and biscuits can be made by the most inexperienced if only the directions on the box are followed in the preparation. As a food of high nutritive properties Carter's oats must be regarded with considerable respect, for although mainly consisting of a readily digested carbohydrate, it contains also a large proportion of vegetable proteid which is well assimilated by the system.

### CALVERT'S SURGEONS' SOAP.

(F. C. Calvert and Co., Manchester.)

This soap has been specially prepared for cleansing the hands of surgeons and the skin of patients before operation, and in addition there are many dermatological uses to which it may be applied. It appears to be a soap admirably adapted to the purposes for which it is intended, and the antiseptic properties of the germicide contained are in no way deteriorated by the combination with the soap basis.

### "VICTORIA VIOLETS" SOAP.

(Evans, Gadd, and Co., Bristol and Exeter.)

We have received samples of the above soap. It appears a carefully manufactured, super-fatted soap, with excellent emollient qualities. It is delicately scented with "Victoria violets" perfume. For toilet purposes it constitutes a most elegant and fragrant "Savon de luxe."

### PERFECT HEALTH FOOD.

(A. J. G. King and Son, 19, Regent Street, W., Swindon.)

This is a specially prepared food for invalids and children. It is well malted, or, in other words, partially digested, and containing nothing that cannot be readily absorbed from the digestive tract, it must be regarded as especially valuable for those whose digestive processes are weak or impaired. Containing as it does a high percentage of wheat germ and phosphates, it should prove a food well adapted to assist the building up of the tissue in growing children or their recovery to the normal after wasting or debilitating illness. To prepare it for use the food should be mixed to a smooth paste with cold milk, and then diluted with further quantities according to the age or requirements of the patient; finally it is boiled for a few minutes.

